# Sandwich teaching improved students' critical thinking, self-learning ability, and course experience in the Community Nursing Course: A quasi-experimental study

**DOI:** 10.3389/fpsyg.2022.957652

**Published:** 2022-08-29

**Authors:** Xiaoyan Cai, Mingmei Peng, Jieying Qin, Kebing Zhou, Zhiying Li, Shuai Yang, Fengxia Yan

**Affiliations:** School of Nursing, Jinan University, Guangzhou, China

**Keywords:** Sandwich teaching, Community Nursing Course, nursing undergraduate, critical thinking, self-learning ability, course experience

## Abstract

The youngest generation of students prefers a more active learning style. Sandwich teaching may suit their learning style by alternating between active individual learning and passive collective learning. Sandwich teaching has been rarely applied to the Community Nursing Course for nursing students, and its teaching effects on this course remain unclear. This study applied Sandwich teaching to the Community Nursing Course for Chinese nursing undergraduates and investigated its effects on students' critical thinking, self-learning ability, course experience, and academic performance. This is a quasi-experimental study with 72 Chinese nursing undergraduates. Students receiving traditional teaching were enrolled in the control group (n = 36), and those who received Sandwich teaching were recruited into the experimental group (n = 36). Both groups received the 12-week, 90-min Community Nursing Course. Our main outcome variable, including students' critical thinking, self-learning ability, and course experience, was assessed by specific questionnaire. The paired t-tests were applied to compare the differences of the same group in the pre-test and the post-test, and the independent-sample t-tests were used to compare the differences between the two groups. We observed that nursing students' critical thinking ability and self-learning ability were significantly improved after receiving Sandwich teaching. Students' course experience of Sandwich teaching was significantly better than that of traditional teaching. The final exam score in the experimental group was not significantly higher than that in the control group. These results suggest that Sandwich teaching in Community Nursing Course improved Chinese nursing undergraduates' critical thinking, self-learning ability, and course experience, but failed in improving academic performance.

## Introduction

The youngest generationof students, widely known as Gen Z, is composed of digital natives preferring an independent learning style with less passive but more visual and kinesthetic learning (Hampton and Keys, 2017; Isaacs et al., 2020). It was reported that Gen Z students want to collaborate often, independently thinking at first and then discussing as a group (Williams, 2019). This kind of learning style should be taken into consideration when educators take classes for them, as learning style is one of the most essential factors influencing the highly individual learning process (Zoghi et al., 2010; Burger and Scholz, 2014). Given that the traditional teacher-centered teaching method has difficulty in suiting Gen Z students' learning style, it is necessary for educators to alter educational practices and shift from being teacher-centered to being learner-centered.

A didactic method called Sandwich teaching takes individual learning into account by consecutively alternating between collective and individual learning phases (Bock et al., 2020). Generally, the collective learning phase is passive and similar to classical lectures, lasting for 20–25 mins within the students' attention span (Bunce et al., 2010). The individual learning phase is active learning by applying the previously gained knowledge to finish precise work assignments such as partner discussions, partner interviews, or small-group work. The alternation of collective and individual learning phases contributes to the consecutive switch between passive learning and active learning. The Sandwich teaching, as a student-centered teaching style, suitable for conducting small-class teaching, has been applied to the teaching of various medical courses (Sun et al., 2014; Shi et al., 2016; Wang et al., 2018; Ling et al., 2019) and was reported to have various benefits on learning outcomes. It was revealed that Sandwich teaching in medical oncology can effectively promote students' acquisition of knowledge and skills and enhance the teaching effect (Zou and Li, 2021). Besides, Sandwich teaching is also demonstrated to improve the theoretical and practical performance of nursing students in different nursing courses (Chen and Li, 2019; Hao and Cui, 2021). For instance, Sandwich teaching in basic nursing theory teaching was to effectively improve Chinese nursing students' judging, thinking, and core ability, as well as teaching quality (Zhang, 2022). However, there is a dearth of evidence available about using Sandwich teaching in the Community Nursing.

The Community Nursing Course is a compulsory course for nursing students in China that aims to build up their knowledge of the system of community nursing and boost their ability to solve the community residents' health problems. This course is of vital importance to cultivate excellent community nurses, especially on the background of Healthy China Action 2030 and the increased aging of the Chinese population. The dominant way of delivering this course is still the traditional in-class lecture, which is passive learning and hardly develops students' competencies. Due to the paucity of evidence on Sandwich teaching in nursing education of the Community Nursing Course and the absence of a formula for how to implement Sandwich teaching in this course, the effects of Sandwich teaching in the Community Nursing Course are not yet completely clear.

Therefore, this study proposed the application of Sandwich teaching in the Community Nursing Course and mainly investigated its effects on nursing students' critical thinking, self-learning ability, and the course experience. Our study is significant in that Sandwich teaching, which is characterized by alternating individual and collaborative learning, takes the youngest nursing students' learning style into consideration, and the implementation process, as well as the results, may provide some useful clues for nursing educators to improve the teaching quality of the Community Nursing Course.

## Materials and methods

### Design

This study adopted a quasi-experimental design using the pre- and post-test and was conducted from 2020 to 2022.

### Setting and participants

This study was conducted at a university in South China. The study sample was composed of 72 third-year nursing undergraduates. Purposive sampling was used. Students who had completed the course *via* the traditional teaching method were enrolled in the control group (n = 36), while those who had not learned Community Nursing were recruited into the experimental group (n = 36) to undergo Sandwich teaching.

### Development of Sandwich teaching

#### Theoretical basis

Sandwich teaching, which originated in the United Kingdom, is a kind of teaching model that alternates and combines theoretical learning and work practice. It was first introduced to medical classroom teaching at Heidelberg University in Germany. This teaching approach highlights the significant role of integrating theory and work practice together through group discussions, cross-learning, and learning reports. With this teaching method, students become the masters of learning *via* preparations before class, discussions, and interactions in the class, while teachers are in the position to guide and assist students in learning during the teaching process.

#### Individual learning design

Teaching resources, such as literature, videos, cases, or website links for learning, were provided for students to carry out individual learning. For instance, students were expected to preview by reading textbooks or watching videos, figure out answers to the questions affiliated with practical cases by referring to related information, and make preparations for group discussions in the class. Specifically, when searching the literature, students were required to analyze the nursing assessment methods, nursing diagnoses, and corresponding nursing measures in the literature, and estimate which one could be feasible in the community health center (Dong et al., 2021).

#### Collaborative learning design

To design collaborative learning, the experience of process-oriented guided inquiry learning (POGIL) pedagogy was drawn from. POGIL is a student-centered active learning approach that works by dividing students into small groups to analyze cases or problems, which was reported to effectively promote learning success with outcomes like course satisfaction (Roller and Zori, 2017) and learning motivation (Smith et al., 2018). Before class, experienced teachers designed distinctive learning materials to guide nursing students to explore new knowledge. When assigned to POGIL activities, participants undertake different roles in the team, including the roles of the leader, recorder, presenter, reflector, checker, and so on. When group members are working together, instructors offer immediate and consecutive feedback according to students' performances in the class (The POGIL Project Team, 2020).

#### Preparation of Sandwich teaching before class

The investigators reviewed the relevant information on the Sandwich teaching methods, integrating information about the definition of a Sandwich teaching method, its application in nursing education teaching, the detailed teaching process, and the teaching form.

Next, the Sandwich teaching plans were set up based on the course contents by nursing professional teachers with more than 5 years of teaching experience. The teaching plans were composed of opening remarks, teaching objectives, and well-designed reasonable questions for discussion (Lin et al., 2020). At this stage, teachers were required to design concise opening remarks to bring up the topic, be familiar with the course content to determine achievable learning objectives, and condense contents into some appropriate questions in accordance with the teaching focus.

Before class, 36 students in the experimental group were stratified by their grade point average (GPA) in the last year and divided into six equal original subgroups, named groups A, B, C, D, E, and F, to avoid obvious differences between the different groups. Also, students in the same group were assigned numbers 1, 2, 3, 4, 5, and 6 as shown in Table 1.

**Table 1:** Original subgroups of panel discussion in the experimental group.

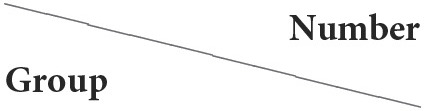	**1**	**2**	**3**	**4**	**5**	**6**	**Question**
Group A	A-1	A-2	A-3	A-4	A-5	A-6	Question A
Group B	B-1	B-2	B-3	B-4	B-5	B-6	Question B
Group C	C-1	C-2	C-3	C-4	C-5	C-6	Question C
Group D	D-1	D-2	D-3	D-4	D-5	D-6	Question D
Group E	E-1	E-2	E-3	E-4	E-5	E-6	Question E
Group F	F-1	F-2	F-3	F-4	F-5	F-6	Question F

### Implementation of Sandwich Teaching in the Community Nursing Course

**Figure 1:**
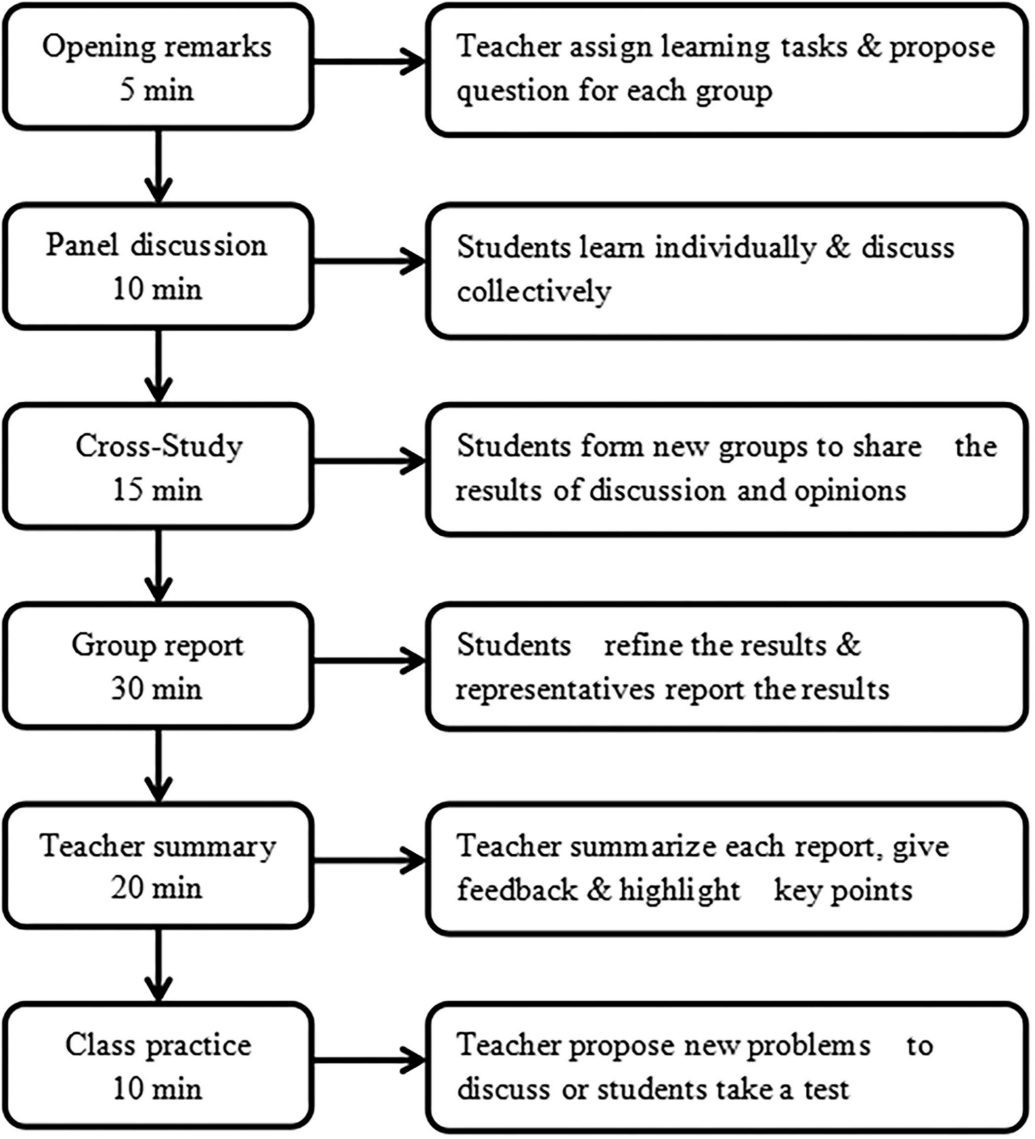
The teaching frame of Sandwich teaching.

The process of Sandwich teaching in a class for the experimental group included the following six steps (Figure 1). Take the community geriatric health care as an example.

Step one (opening remarks, 5 mins): Students were divided into six groups in the classroom; the teacher started class with the following case designed in line with the teaching context to capture students' attention and put forward different questions for each group to discuss.

Case: Mr. Li is a 65-year-old patient with stroke sequelae including a right side of palsy and the declined language function. After 4 months of hospitalization, he was discharged, nearly spent all his time in the bed, and had few opportunities to communicate with others except for family members.

Questions:

1. What are the nursing problems with this patient?
2. What aspects of health guidance should be given to this patient by community nurses?
3. How to ensure this patient's safety at home?
4. How to guide this patient to do the rehabilitation training at home?
5. How to offer psychological support to this patient?
6. What healthy dietary guidance should be given to this patient?

Step two (panel discussion, 10 mins): Nursing students in each group learned individually with learning materials (such as textbook) at first and then learned collectively through discussing to reach a consensus on the issues. Each group mainly discussed one of the assigned questions.

Step three (cross-study, 15 mins): Students in a group separately went to the other five groups to form a new group. For example, A-1, B-1, C-1, D-1, E-1, and F-1 formed a new group, while A-2, B-2, C-2, D-2, E-2, and F-2 formed a new group, as shown in Table 2. Each student was required to share the results of the discussion with new group members, expressed their opinions, and listen to others' opinions, so that they can be fully familiar with the question and participated in this section.

**Table 2:** New subgroups for cross-study in the experimental group.

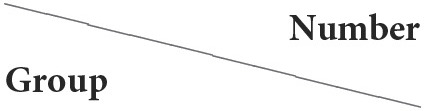	**Group 1**	**Group 2**	**Group 3**	**Group 4**	**Group 5**	**Group 6**	**Question**
A	A-1	A-2	A-3	A-4	A-5	A-6	Question A
B	B-1	B-2	B-3	B-4	B-5	B-6	Question B
C	C-1	C-2	C-3	C-4	C-5	C-6	Question C
D	D-1	D-2	D-3	D-4	D-5	D-6	Question D
E	E-1	E-2	E-3	E-4	E-5	E-6	Question E
F	F-1	F-2	F-3	F-4	F-5	F-6	Question F

Step four (group report, 30 mins): Students went back to the original group, integrated new results in cross-study with the previous results in panel discussion, and summarized and wrote down the final results; each group sent a representative to report the final results for 4–5 mins.

Step five (teacher's summary, 20 mins): After reporting, the teacher comprehensively and deeply analyzed the case and the questions, guided the students to think, answered the questions involved in the case, pointed out the advantages and disadvantages of each group's report as feedback, supplemented the results, summarized the teaching content, and highlighted the key points of the teaching content by combining the case and the theory in the textbook.

Step six (class practice, 10 mins): Teacher put forward new questions related to the case for students to cogitate and discuss, so as to deepen students' understanding of teaching content and foster their ability to apply knowledge to solve practical problems.

Sandwich teaching was delivered to the experimental group once a week, 90 mins per week, lasting for 12 weeks. The control group received traditional lecture teachings in which the teachers organized students to give concentrated lectures to deliver professional knowledge. The teaching requirements and teachers were the same.

### Data collection

Quantitative data on students' critical thinking ability and self-learning ability were collected to better understand the effects of Sandwich teaching by using special scales at the beginning and the end of the course. Besides, participants' age, gender, average grade point average (GPA), final exam scores for this course, and course experience were recorded at the end of the semester.

#### Critical thinking disposition

The Critical Thinking Disposition Inventory of Chinese Version (CTDI-CV) developed by Pang et al. (2004) was adopted to determine students' critical thinking ability. This scale included seven dimensions, which were truth-seeking, open-mindedness, analysis ability, systematization ability, self-confidence in critical thinking, inquisitiveness, and cognitive maturity, with a total of 70 items. Each dimension consisted of 10 items and used the six-point Likert scale from 1 (strongly disagree) to 6 (strongly agree). The total score ranged from 70 to 420. A total score of <210 indicates a negative critical thinking tendency, a total score of 210–280 indicates a moderate critical thinking tendency, a total score of 281–349 indicates a positive critical thinking tendency, and a total score of 350–420 indicates a strong critical thinking tendency. Each dimension that scored <40 represents a weak disposition, each one that scored 40–49 represents a positive disposition, and each one that scored 50–70 represents a strongly positive disposition. The Cronbach's alpha values ranged from 0.54 to 0.77 for the seven dimensions, and the value was 0.90 for the total scale.

#### Self-learning ability

The self-learning ability scale developed by Zhang and Li (2009) was employed to anonymously evaluate nursing students' self-learning ability. This scale has 30 items measuring four dimensions, including self-management (11 items), learning motivation (8 items), information management (6 items), and learning cooperation (5 items). Each item uses a five-point Likert score ranging from 1 (completely disagree) to 5 (completely agree). The total score ranges from 30 points to 150 points, and a higher score stands for a better self-learning ability. The internal consistency reliability of the scale was 0.822, and the split-half reliability was 0.788.

#### Course experience

The Course Experience Questionnaire (CEQ) was widely employed to determine students' perception of teaching quality (Ramsden, 1991). The Chinese version of the CEQ, which was revised and applied to medical students by Yan et al. (2019), was adopted in our study to investigate learners' course experience. The adopted CEQ contains 25 items divided into three domains, including class quality and harvest (13 items), good teaching (10 items), and the appropriate workload (2 items), using a five-point Likert score ranging from 1 (completely disagree) to 5 (completely agree). The internal consistency reliability of the CEQ was 0.962, and the split-half reliability was 0.861.

#### Final examination

A theoretical examination was used to evaluate students' mastery of knowledge at the end of the course. The exam included single-choice questions, multiple-choice questions, brief-answer questions, case analysis questions, and noun explanations so as to comprehensively assess students' mastery of theoretical knowledge, analysis, and application ability. The total score of the final exam was 100 points.

### Data analysis

The demographic characteristics and changes in scores were described using the mean and standard deviation. Paired sample t-tests were employed to determine differences in critical thinking ability and self-learning ability between the pre-test and the post-test. *P* < 0.05 indicated statistical significance for all tests.

## Results

### Characteristics of students

A total of 72 third-year nursing undergraduates aged 19–23 years participated in this study. The mean age was 21.17±1.06 for the experimental group and 21.13±1.17 for the control group. There were 11 (30.6%) males and 25 (69.4%) females in the experimental group and 9 (25.0%) males and 27 (75.0%) females in the control group. The average GPA was 3.08 ± 0.59 for the experimental group and 3.12 ± 0.67 for the control group. The experimental group did not significantly differ from the control group in terms of age (*P* = 0.598), gender (*P* = 0.793), and GPA (*P* = 0.819).

### Critical thinking ability

As presented in Table 3, in the pre-test, the participants in both groups scored <280 points in CTDI-CV, and the scores in almost all domains were below 40, indicating that the students' critical thinking ability was weak. After the course, the students in the experimental group scored >280 in the critical thinking ability while those in the control group still scored <280. The results showed that the experimental group had a significant improvement in the total CTDI-CV score after receiving Sandwich teaching, whereas the control group did not. As for the domains of CTDI-CV, significant differences were noted in the systematization ability and cognitive maturity between the two groups after the course, and the students in the experimental group achieved significant improvements in these two domains.

**Table 3:** Comparison of CTDI-CV and its dimension score in pre- and post-test and between two groups (M ± SD).

**Domains**	**Group**	**Pre-test**	**Post-test**	***P* value^b^**
Truth-seeking	CG	35.75 ± 8.94	35.03 ± 7.23	0.693
	EG	34.17 ± 8.90	37.56 ± 7.77	0.037
	*P* value^a^	0.454	0.157	–
Open-mindedness	CG	36.58 ± 5.67	38.58 ± 6.15	0.106
	EG	38.64 ± 6.50	40.78 ± 6.34	0.114
	*P* value^a^	0.157	0.140	–
Analytical ability	CG	39.00 ± 5.34	40.06 ± 5.21	0.215
	EG	39.08 ± 6.72	40.56 ± 6.03	0.236
	*P* value^a^	0.954	0.708	–
Systematization ability	CG	36.00 ± 4.45	35.81 ± 4.64	0.832
	EG	35.64 ± 4.98	39.75 ± 5.58	**<0.001**
	*P* value^a^	0.746	**0.002**	–
Self-confidence in	CG	37.14 ± 8.32	38.25 ± 7.06	0.389
Critical thinking	EG	38.11 ± 7.49	38.83 ± 8.76	0.688
	*P* value^a^	0.604	0.757	–
Inquisitiveness	CG	39.42 ± 5.90	41.36 ± 6.02	0.131
	EG	41.11 ± 8.02	41.56 ± 7.55	0.757
	*P* value^a^	0.311	0.904	–
Cognitive maturity	CG	36.67 ± 9.46	36.31 ± 7.46	0.832
	EG	36.58 ± 10.45	41.78 ± 7.63	**0.019**
	*P* value^a^	0.972	**0.003**	–
Total score	CG	260.56 ± 25.53	265.39 ± 28.35	0.182
	EG	263.33 ± 25.49	280.81 ± 35.60	**0.002**
	*P* value^a^	0.646	**0.046**	–

CTDI-CV, Critical Thinking Disposition Inventory of Chinese Version; EG, experimental group; CG, control group; M, mean; SD, standard deviation.

a The independent-sample t-test;

b The paired-sample t-test.

The bold values indicate the statistically significant differences of value of *P* < 0.05.

### Self-learning ability

As shown in Table 4, the changes in total scores and all dimensions of self-learning ability in the experimental group were significant (*P* **<** 0.05) while those in the control group were not. The score of the dimension of the learning cooperation ability in the Sandwich teaching group was significantly higher than that of the control group in the post-test, while this was not the case for other dimensions of self-learning ability.

**Table 4:** Comparisons of self-learning ability in pre- and post-test and between two groups (M ± SD).

**Domains**	**Group**	**Pre-test**	**Post-test**	***P* value^b^**
Learning motivation	CG	30.78 ± 4.14	31.89 ± 4.71	0.166
	EG	30.42 ± 4.68	32.31 ± 4.43	**0.025**
	*P* value^a^	0.730	0.700	–
Self-management ability	CG	40.69 ± 4.73	41.44 ± 4.29	0.419
	EG	40.36 ± 4.76	43.03 ± 4.31	**0.001**
	*P* value^a^	0.767	0.122	–
Learning cooperation	CG	17.67 ± 2.45	17.64 ± 2.09	0.957
Ability	EG	17.42 ± 2.44	18.89 ± 2.27	**0.002**
	*P* value^a^	0.666	**0.017**	–
Information quality	CG	21.97 ± 3.38	22.14 ± 3.15	0.776
	EG	21.94 ± 3.01	23.17 ± 2.37	**0.019**
	*P* value^a^	0.971	0.122	–
Total Score	CG	111.11 ± 12.02	113.11 ± 11.90	0.350
	EG	110.14 ± 12.59	117.39 ± 11.41	**<0.001**
	*P* value^a^	0.739	0.124	–

EG, experimental group; CG, control group; M, mean; SD, standard deviation.

a The independent-sample t-test;

b The paired-sample t-test.

The bold values indicate the statistically significant differences of value of *P* < 0.05.

### Course experience

Table 5 shows the mean values of the total scores and three domains of the CEQ. The results showed significant differences in the total score, domains of classroom quality and harvest, and appropriate workload, which scored higher in the experimental group. The score of good teaching in the experimental group was higher than that in the control group; however, there was no significant difference between the two groups.

**Table 5:** Comparisons of students' course experience between two groups after the course (M ± SD).

**Domains**	**CG**	**EG**	***P* value**
Class quality and harvest	40.42 ± 11.98	46.67 ± 9.85	**0.018**
Good teaching	36.06 ± 7.14	38.86 ± 6.23	0.080
Appropriate workload	4.83 ± 1.46	5.72 ± 2.00	**0.036**
Total Score	81.31 ± 18.53	91.25 ± 16.09	**0.018**

EG, experimental group; CG, control group; M, mean; SD, standard deviation. The bold values indicate the statistically significant differences of value of *P* < 0.05.

### Final exam score

The maximum and minimum scores in the experimental group were 96 and 64 while those in the control group were 96 and 60. The score of the experimental group (83.47 ± 9.35) was not significantly better than that of the control group (80.58 ± 9.49, *P* = 0.197).

## Discussion

To the best of our knowledge, this is the first study that used Sandwich teaching in the Community Nursing Course for Chinese full-time nursing undergraduates. Our results revealed that Sandwich teaching exerted some positive effects on third-year undergraduate nursing students in China and was feasible for delivering the Community Nursing Course. As demonstrated in a previous study, Sandwich teaching increased students' learning gain, engagement in learning activities, and satisfaction with teaching (Katsioudi and Kostareli, 2021).

### Sandwich teaching improved students' critical thinking ability

The significantly higher score of CTDI-CV in the Sandwich teaching group implied this teaching method may improve the critical thinking ability of nursing students. Our results showed that Sandwich teaching could be a useful strategy in developing students' systematization ability and cognitive maturity, which is consistent with the findings of a previous study by Zhang (2014). Compared to the control group, only two domains (the systematization ability and cognitive maturity) significantly scored higher in this study, whereas six dimensions (except for the systematization ability in the Sandwich teaching group) scored significantly higher in the study conducted by Bao and Hai (2016).

Since the teachers, total class hours, and textbook were the same in both groups in our study, the significantly higher score of the critical thinking ability may be because Sandwich teaching reinforced active learning and helped cultivate various abilities related to critical thinking in some ways. Firstly, group work such as panel discussions and cross-study required students to extensively think, identify, and analyze problems, which involved critical thinking (Geist et al., 2015). Every student in the Sandwich teaching group had the opportunity to participate in the discussion together, which means Sandwich teaching has obvious advantages in harnessing students' learning enthusiasm, training their language organizational ability, and cultivating their ability to analyze and solve problems (Zhong et al., 2019). Additionally, this study adopted active learning strategies, including case studies, group problem-solving, and discussions, to strengthen students' active involvement in learning (Von Colln-Appling and Giuliano, 2017) and thus contribute to the promotion of critical thinking (Nelson, 2017). Moreover, the student–student and student–teacher interactions during the learning process might expand the learners' scope of cognition, make them alert to accept multiple solutions and carefully make judgments; in the meanwhile, when students made organized and targeted efforts to solve problems in the process of completing group tasks, their ability to systematize was gradually developed.

### Sandwich teaching promoted students' self-learning ability

Our study showed that Sandwich teaching exerted positive effects on students' self-learning abilities. There were significant differences in the dimensions of self-management, learning motivation, information management, and learning cooperation. As Lin et al. (2020) reported, Sandwich teaching was conducive to improving students' learning initiative, and the learning initiative score of the students in the experimental group was significantly higher than that of students in the control group. In this study, learning materials were beneficial to create practical situations that may capture the students' attention and inspire them to engage in learning activities; at the same time, this teaching method increased the interaction between teachers and students, as well as among students, which conducive to mobilize students' initiative of learning and improve their abilities of self-learning, thinking, and exploring new knowledge. As a result, students' learning motivations were promoted.

Apart from that, Sandwich teaching was task-driven by assigning learning tasks to every student, and students were driven to manage their time to study in advance and found relevant information to support their opinions during classroom discussions. Eventually, students' abilities of self-management and information management were significantly fostered. Furthermore, Sandwich teaching strengthened students' learning cooperation ability through group work, as joined efforts were needed to turn the results of panel discussion into a complete group report. As concluded by Zhong et al. (2019), Sandwich teaching had obvious advantages in cultivating learners' team spirit.

### Sandwich teaching improved students' course experience

This study revealed that Sandwich teaching improved students' course experience. The significantly higher course experience score in the Sandwich teaching group implied this teaching approach may enhance students' engagement and satisfaction with the teaching of this course. A previous study provided strong evidence that Sandwich teaching enhanced not only students' engagement but also their satisfaction with teaching (Katsioudi and Kostareli, 2021). Moreover, students receiving Sandwich teaching were significantly satisfied with the teaching preparation, teaching process, teaching ability, and teaching outcomes in the experimental group compared to their counterparts receiving traditional teaching (Yang and Liu, 2015).

In our study, students in the experimental group thought that Sandwich teaching imposed an appropriate workload. This is likely because the problems designed for Sandwich teaching were neither too simple nor too difficult, and they were closely related to practical nursing work to maintain students' interest in independent learning and discussion (Lin et al., 2020). In addition, discussing or interacting with counterparts and teachers exposed students to multiple opinions or views; consequently, their understanding of knowledge was deepened, and their cognitive scopes were expanded. Thus, students in the Sandwich teaching group spoke highly of the class quality and harvest and scored significantly better than those in the control group.

However, in terms of good teaching, the students in the experimental group did not score significantly higher than those in the control group. This result is in line with a previous finding that no significant difference between the two groups' course evaluations (Lin et al., 2020). This may be due to the teachers' dedication to delivering the course, which led to good teaching effects on both groups. Generally, Sandwich teaching improved students' course experience by providing them with an interactive learning experience.

### Sandwich teaching did not significantly improve students' academic performances

We found that the final exam score in the Sandwich teaching group was higher than that in the classical lecture group although the difference was not statistically significant. This finding was not completely consistent with the results of previous studies. For example, a study conducted on 168 Chinese nursing undergraduates revealed that the score of the final theory test in the Sandwich teaching group (90.84 ± 3.32) was significantly higher than that in the control group (88.72 ± 3.64) (Yang and Liu, 2015). Likewise, Long et al. (2016) and Lin et al. (2020) reported that the theoretical results of nursing students receiving Sandwich teaching were statistically better than those of students receiving classical lectures. Besides, another study reported that the total test score and the scores of multiple-choice questions, discussion questions, and case analysis questions in the Sandwich teaching group were significantly higher than those of the same tests in the control group (Bao and Hai, 2016). Panel discussions and cross-studies may lead to active learning, foster students' understanding of knowledge, and help students to achieve better academic performances.

Our finding of no significant difference between the two groups may be attributed to some reasons. Firstly, the difficulty of the Community Nursing Course was acceptable for the majority of students; so, they were able to master the main course contents. Secondly, the Chinese students we included in our study were hardworking enough to pass the exam. Thirdly, the curriculum executors may not be proficient enough in using Sandwich teaching at the beginning of the implementation process. In Sandwich teaching, every part of the content and the time need to be carefully designed and arranged to make each part closely linked, interlocked, and deepened step-by-step, and the teachers should effectively guide and control the process of students' discussion in the classroom, which put forward higher requirements for the teacher's ability to control the classroom rhythm and design a scientific, reasonable, effective, and meticulous teaching plan (Long et al., 2016). In addition, this course when delivered by using the same teaching method might not consecutively stimulate and support students' learning interests in the long term.

To fully demonstrate the effect of Sandwich teaching on learning outcomes, combining the Sandwich teaching approach with other diverse teaching methods may be a promising attempt. It was found that role conversion combined with the Sandwich teaching method in obstetrics and gynecology nursing undergraduate education could improve the learning outcomes of knowledge acquisition and the comprehensive ability and promote students' satisfaction with the teaching model (Wang et al., 2017). Moreover, Sandwich teaching combined with situational simulation teaching was demonstrated to have positive effects on the nursing technology training of traditional Chinese medicine (Lyu, 2021).

Overall, the innovative point of this study was that Sandwich teaching was adopted in the Community Nursing Course for Chinese nursing undergraduates. Our findings implicated nursing educators to apply Sandwich teaching in nursing education programs in terms of cultivating students' critical thinking, self-learning ability, and ameliorating course experience.

### Limitations and future directions

This study has some drawbacks. Firstly, we only investigated the effects of Sandwich teaching on the Community Nursing Course in a Chinese university, so the universality of our results should be taken into consideration. Therefore, multicentered and large-sample studies conducted in different cultural settings are needed to confirm our findings. Additionally, we investigated the effect of Sandwich teaching on the theoretical teaching of nursing education but did not investigate its effect on nursing students' practical skills. Moreover, the quasi-experimental study design could not avoid all influences exerted by confounders. Thus, it is essential to examine our findings with more rigorous randomized controlled trials in the future.

## Conclusion

This study demonstrated that Sandwich teaching played a significant role in improving Chinese nursing students' critical thinking, self-learning ability, and course experience, but failed in improving academic performance. Our findings suggested that nursing educators implement Sandwich teaching to adapt to the youngest generation of nursing students' learning styles so as to facilitate their learning. Future studies are needed to further explore the learning effectiveness of Sandwich teaching.

## Data availability statement

The raw data supporting the conclusions of this article will be made available by the authors, without undue reservation.

## Ethics statement

The studies involving human participants were reviewed and approved by Institutional Review Board of Jinan University. The patients/participants provided their written informed consent to participate in this study.

## Author contributions

XC and MP contributed to conception and design of the study. JQ and KZ collected the data. ZL performed the statistical analysis. XC wrote the first draft of the manuscript. MP, SY, and FY wrote sections of the manuscript. All authors contributed to manuscript revision, read, and approved the submitted version.

## Funding

The authors declare that this study received funding from Medicine School at Jinan University where this study was conducted as a teaching reform project (Grant Number: 2021YXJG002), the 23^rd^ Batch of Teaching Reform Projects of Jinan University, Guangdong Province Undergraduate University Teaching Quality and Teaching Reform Project Construction Project in 2021. The funder was not involved in the study design, collection, analysis, interpretation of data, the writing of this article, or the decision to submit it for publication.

## Conflict of interest

The authors declare that the research was conducted in the absence of any commercial or financial relationships that could be construed as a potential conflict of interest.

## Publisher's note

All claims expressed in this article are solely those of the authors and do not necessarily represent those of their affiliated organizations, or those of the publisher, the editors and the reviewers. Any product that may be evaluated in this article, or claim that may be made by its manufacturer, is not guaranteed or endorsed by the publisher.
